# Editorial: Alternative treatments to classical antidepressants in treatment-resistant depression

**DOI:** 10.3389/fpsyt.2023.1306117

**Published:** 2023-11-06

**Authors:** Georgios Mikellides, Theodoros Koutsomitros, Olympia Evagorou, Nikolaos Gkouvas, Kuan-Pin Su, Panayiota Michael

**Affiliations:** ^1^Cyprus rTMS Centre, Larnaca, Cyprus; ^2^Department of Cognitive Neuroscience, Faculty of Psychology and Neuroscience, Maastricht University, Maastricht, Netherlands; ^3^Medical School, University of Nicosia, Nicosia, Cyprus; ^4^Greek rTMS Clinic, Medical Psychotherapeutic Centre (IΨK), Thessaloniki, Greece; ^5^Medical Psychotherapeutic Centre, Institute of Psychotherapy, Thessaloniki, Greece; ^6^Hellenic Psychiatric Association, Athens, Greece; ^7^Mind-Body Interface Research Center (MBI-Lab), China Medical University Hospital, Taichung City, Taiwan; ^8^An-Nan Hospital, China Medical University, Tainan, Taiwan

**Keywords:** treatment-resistant depression, serotonergic psychedelics, ayahuasca, ketamine, oral esketamine, transcranial magnetic stimulation, transcranial electric stimulation

Depression is a serious mental health disorder that affects people worldwide. Although antidepressants are still commonly used to treat depression, one-third of individuals are unresponsive to medication, resulting in what is known as treatment-resistant depression (TRD). Hence, alternative treatment options are urgently needed for those who are unresponsive to conventional treatments. Within this Research Topic, five papers delve into alternative treatments for depression, investigating the effects and mechanisms of serotonergic psychedelics (like ayahuasca), oral esketamine, the combination therapy of ketamine and transcranial magnetic stimulation (TMS) and transcranial electric stimulation (TES).

A review of published articles was conducted by Husain et al. to evaluate the antidepressant mechanism of action of serotonergic psychedelics. Their findings suggest that serotonergic psychedelics impact depression through multiple factors, such as stimulation of serotonin receptors, promotion of neurogenesis and neuroplasticity, modulation of the immune system, changes in brain connectivity, and psychological effects. The psychoactive effects of serotonergic psychedelics are believed to occur through their agonism of the serotonin (5-HT) receptor 2A, leading to downregulation of these receptors and changes in neuronal excitability in the cortex. While the 5-HT1A receptor may also play a role in their antidepressant effect, more research is required to determine the specific receptors involved for each psychedelic drug. By indirectly activating glutamate networks, serotonergic psychedelics impact prefrontal areas and increase brain-derived neurotrophic factor (BDNF), which supports neuron growth and neuroplasticity. Furthermore, the authors discuss the relationship between depression and inflammation, with evidence indicating that patients with major depressive disorder (MDD) have elevated inflammatory markers. Additionally, neuroimaging and neurophysiology studies have shown that psychedelics may have potential as an antidepressant treatment by altering brain connectivity patterns and emotional processing. Finally, the authors suggest that psychedelics may assist individuals with depression by facilitating transitions away from maladaptive thought patterns, restoring emotional responsiveness, and inducing an emotional release. Factors such as ego dissolution, enhanced sense of connection with others, and mystical experiences may also contribute to the antidepressant effect of psychedelics.

In a randomized controlled trial (RCT) with 72 participants, consisting of 28 patients with TRD and 44 healthy controls, de Sousa et al. investigated how a group of acute parameters—emotional (depressive symptoms), cognitive (psychedelic experience), and physiological (salivary cortisol) moderated certain important molecular biomarkers for MDD, serum brain-derived neurotrophic factor (BDNF), serum cortisol (SC), serum interleukin 6 (IL-6), plasma C-reactive protein (CRP), and salivary cortisol awakening response (CAR) 2 days post intervention. Both patients and controls received a single oral liquid dose of either ayahuasca or placebo. Results showed that during the ayahuasca dosing session, a larger acute decrease in depressive symptoms was associated with significantly higher levels of SC in patients. Furthermore, in patients who demonstrated a greater clinical response with respect to the reduction of depressive symptoms, lower changes in salivary cortisol levels during the ayahuasca intervention were linked with higher levels of serum BDNF. This effect was observed in patients with depression, compared to healthy controls, and particularly in those with a more significant clinical response. These results have implications for future research exploring the biological and psychological changes resulting from psychedelic therapies.

In recent years, there has been particular interest in the antidepressant effects of ketamine in patients with TRD. Ketamine is a racemic mixture of two enantiomers, R- and S-ketamine, with the latter also known as esketamine. Esketamine is used as a standalone treatment for TRD and MDD. In their qualitative study, Breeksema et al. aimed to explore the perspectives and experiences of TRD patients who received repeated oral esketamine treatment in an “off-label” manner (either inpatient, outpatient, or at-home treatment). The study involved conducting in-depth interviews with 17 patients who had completed a 6-week, twice-weekly esketamine treatment program and continued to use the treatment at home for 6 months. The interviews focused on the participants' viewpoints, expectations, and experiences with esketamine treatment. The study identified several key themes, including overwhelming experiences, inadequate preparation, letting go of control, the influence of mood states on sessions, the importance of presence and emotional support, and the significance of supportive settings. The findings suggested that the quality of care in esketamine treatment depends not only on the pharmacological intervention but also on the specific elements of set and setting. These elements include preparing patients, offering reassurance, minimizing anxiety, instilling confidence, promoting agency, and creating a warm, comfortable, silent, and private environment with physical, interpersonal, and empathetic professional or informal support. The study also highlighted patients' attempts to let go and give in to the occasionally overwhelming experience vs. trying to maintain control. Factors such as preparation and education level, physical and emotional support, and the session's setting impacted patients' ability to let go, influencing their mood and anxiety about future sessions. Future research should consider these findings to enhance the quality of patient care in (es) ketamine treatments.

Alongside ketamine, TMS has been another alternative treatment option for depression that has garnered significant attention in recent decades. Furthermore, there is growing evidence to suggest that combining TMS and ketamine may be an effective approach for treating TRD. A case report of Elkrief et al. investigated the effectiveness of a combination rTMS and ketamine protocol in a 43-year-old male patient suffering from bipolar TRD. Initially, the patient received separate treatments of HF-rTMS and IV ketamine, but these had limited results. Subsequently, an intensive treatment that combines rTMS and ketamine is offered for 2 weeks. The combined protocol involves the patient receiving three sessions of standard intermittent theta burst stimulation (iTBS) over the left DLPFC on each weekday afternoon, with 50 min between each session (30 sessions in total). Additionally, the patient receives six sessions of IV ketamine protocol on three mornings per week. After a positive response to the initial combined treatment, a 10-week consolidation phase was implemented. During this phase, the patient received 2 days of rTMS per week, with three sessions per day spaced 50 min apart for the first 6 weeks, followed by 1 day per week for the remaining 4 weeks, for a total of 48 treatment sessions. Each rTMS session started with a 2-min continuous TBS treatment, followed directly by the iTBS protocol. The patient also received intramuscular (IM) racemic ketamine, once a week for the first 6 weeks and then once every 2 weeks for a total of eight sessions. During the consolidation phase, the patient's clinical condition continued to improve, ultimately resulting in complete and sustained remission. These findings indicate the possible application of combined TMS and IV ketamine therapy and emphasize the requirement for further investigations.

Furthermore an open-label naturalistic study, Koutsomitros et al., assessed the practical feasibility, tolerability and clinical effectiveness of home-administered transcranial direct current stimulation (tDCS) with asynchronous remote supervision, in the treatment of depression. Over the course of 3 weeks, 40 patients with depression received psychotherapy and half of this group also received daily bi-frontal tDCS stimulation of the dorsolateral prefrontal cortex. These patients received tDCS for 30 minutes per session with the anode placed over F3 and the cathode over F4, at an intensity of 2 mA for 21 consecutive days. They measured patients' level of depression symptoms at four time points using the Beck Depression Inventory, before treatment and at 1-week intervals throughout the treatment period. They monitored practical feasibility such as daily protocol compliance and tolerability including side effects, with the PlatoScience cloud-based remote supervision platform. Out of the 20 patients in the tDCS group, 90% were able to comply with the protocol by not missing more than three of their assigned sessions, and none dropped out of the study. No serious adverse events were reported, with only 14 instances of mild to moderate side effects and two instances of scalp pain rated as severe, out of a total of 420 stimulation sessions. Patients in the tDCS group showed a significantly greater reduction in depression symptoms after 3 weeks of treatment, compared to the treatment as usual (TAU) group [t_(57.2)_ = 2.268, *p* = 0.027]. The tDCS group also showed greater treatment response (50%) and depression remission rates (75%) compared to the TAU group (5% and 30% respectively). These findings provide a possible indication of the clinical effectiveness of home-administered tDCS for the treatment of depression, and its feasibility and tolerability in combination with asynchronous supervision.

In conclusion, this Research Topic investigates alternative treatment options for individuals who exhibit TRD. The papers primarily explore the use of the use of serotonergic psychedelics, oral esketamine, and a combination therapy of ketamine and TMS for the treatment of depression. The findings of these studies suggest that serotonergic psychedelics exert antidepressant effects through multiple factors, while the quality of care in esketamine treatment depends on specific elements of set and setting in addition to the pharmacological intervention. Moreover, combining ketamine with TMS therapy might provide a synergistic effect in treating bipolar depression. The findings of these studies have significant implications for future research and the development of alternative treatments for depression.

## Author contributions

GM: Writing—original draft. TK: Writing—review & editing. OE: Writing—review & editing. NG: Writing—review & editing. K-PS: Writing—review & editing. PM: Writing— original draft.

